# Dancing My Age: Emotions, Interactions, and Bodily Sensations

**DOI:** 10.3389/fspor.2022.804888

**Published:** 2022-03-08

**Authors:** Satu Heikkinen, Monika Wilinska

**Affiliations:** ^1^Department of Social and Psychological Studies, Karlstad University, Karlstad, Sweden; ^2^Department of Social Work, School of Health and Welfare, Jönköping University, Jönköping, Sweden

**Keywords:** dance, older people, social interaction, felt age, emotionally perceived age, aging, sociology of emotions

## Abstract

While there is a growing body of research on the social aspects of older people's dance, studies focusing on emotions are rare. In this study, we use an interactionist sociological perspective to examine the role of emotions in older social dancers' experiences in Sweden. Through qualitative interviews with 29 active or previously active dancers, we found that their experiences of emotional energy and experiences of flow override concerns of age and aging. Age, however, did become significant as the age differences at dance events could bring forth feelings of alienation associated with feeling old. In addition, cultural and gendered norms of appropriate age differences between dancing partners produced shame and pride as well as feelings of being either old or young. Moreover, certain bodily experiences were interpreted in terms of age. Overall, the study contributes to the discussions of the complexity of subjective experiences of age by highlighting its emotional aspects through social partner dancing.

## Introduction

“You get a special feeling in your body when dancing”, a woman, aged 65, said to explain why dancing makes her feel young. What does this mean? Does she identify as young because she is fitter than many others of the same age? Does she associate dance with youth and believe she may be viewed as young because she dances? Feeling young or old, younger or older, or talking about “agelessness” and that age does not matter are, however, not restricted to dance but occur frequently in everyday situations. For instance, a body of research concerning subjective age and age identity testifies to a great variety of uses of age categories to express self-identity and sense of belonging to various groups (e.g., Kastenbaum et al., 1972; Nikander, 2000, 2009; Kaufman and Elder, 2002; Diehl et al., 2014; Pinquart and Wahl, 2021). In this article, we apply the concept of emotionally perceived age as we focus on feelings related to subjective experiences of age (see also Alfredsson-Olsson and Heikkinen, 2019). By focusing on emotions and age in heterosexual older people's dance in Sweden, we highlight how age evokes many different feelings and how age becomes an emotion in various dance interactions and situations. Thus, the two main research questions guiding this article are: How are emotions evoked in relation to age in dancing? When and how is age described as a feeling?

In this article, we lean on the interactionist sociological understanding of emotions which conceives of emotions in terms of social phenomena arising in the context of social interactions and relations (Hochschild, 1983; Collins, 2004). Emotions are therefore seen as both individually experienced and collectively shared, including bodily, cultural, and social aspects (Woodward, 2009; Bericat, 2016). While research on the social formation of emotions is vast, we know little about this process in the context of older people's dancing, i.e., partner dance. The sociology of emotion framework is combined with an interactionist perspective on age. Older people's partner dance involves interaction in several dimensions: the interaction between two individuals, between couples, and between all others present at the dance event. Norms related to the dance context are intertwined with more general cultural norms. Last but not least, relationships between women and men are accentuated in heterosexual older people's partner dance. All these aspects create a unique social situation that may be filled with and may produce a myriad of emotions. To examine how both age and emotions are interactionally and socially construed in partner dance, we first review the existing literature on dance, emotions and age before detailing our theoretical framework. We then describe our methods and present our analysis organized in three themes. Lastly, we discuss our key research findings and their relevance for understanding the interplay of age and emotions in partner dancing.

## Previous Research: Dance, Emotions, and Old Age

Research on older people's dancing has been dominated by a medical perspective with its focus on health and interventions (see, e.g., reviews by Keogh et al., 2009; Connolly and Redding, 2010; Guzmán-García et al., 2013). Studies show that dance enhances life quality (Brustio et al., 2018), life satisfaction (Cruz-Ferreira et al., 2015), and psychological wellbeing (Hui et al., 2009), thus indicating that emotions play an important role in dancing in old age. Fewer studies have focused on social aspects even though this is a growing field since leisure activities are highlighted as vital for active aging (Brown et al., 2008; Stevens-Ratchford, 2016). Also research on the social aspects of older people's dancing demonstrates that dancing may generate a broad range of positive emotions, such as a sense of belonging, intimacy, self-esteem, pride and joy as well as negative emotions, such as frustration when turned down and grief when the body fails (Cooper and Thomas, 2002; Nadasen, 2008; Paulson, 2009; Stevens-Ratchford, 2016; Schneider and McCoy, 2018; Alfredsson-Olsson and Heikkinen, 2019). Several of these studies touch on emotions in connection with respondents' accounts of the meaning of dance to them (Cooper and Thomas, 2002; Stevens-Ratchford, 2016; Araujo and Rocha, 2019) and some articles also refer to emotions within a serious leisure perspective (Stevens-Ratchford, 2016; Schneider and McCoy, 2018).

However, few studies have *focused* on emotions as a social dimension in dance among older persons. Two important exceptions taking an interactionist sociology of emotion perspective, study the interplay between positive and negative emotional energy in dancing (Alfredsson-Olsson and Heikkinen, 2019; Heikkinen and Alfredsson-Olsson, 2020). These two studies show that participants not only seek positive emotional energy and experience positive emotions, but also that negative emotional energy has an important function in mustering the energy to continue dancing despite adversity. Heikkinen and Alfredsson-Olsson (2020) argue that the interaction of positive and negative emotional energy among older social dancers provides further understanding of perseverance in serious leisure (Stebbins, 1992) and explicates why a certain effort is required to experience flow. A peripheral but interesting result in Alfredsson-Olsson and Heikkinen (2019) is that the participants mentioned both feeling young and old when dancing, which the authors refer to as emotionally felt age or emotionally perceived age. A third important exception investigates how older people at a senior center talked about feelings of age in connection with activities, including dance (Choi et al., 2021). The study focuses on felt age and its correlation with physical, functional, and mental health among older people. Participating in activities such as dancing was one of the main reasons for feeling younger. In contrast, those who felt older than their chronological age explained that they were less healthy and suffered pain, which in turn limited their opportunities to participate in such activities.

Alfredsson-Olsson and Heikkinen (2019) and Choi et al. (2021) signal the significance of emotions related to how age is experienced when dancing. However, the studies use different approaches toward emotions. Choi et al. (2021) apply the concept of felt age synonymously with the concepts of subjective age and age identity, pointing to a tradition of viewing feelings of age as stable and decontextualized (Montepare, 2009; Nikander, 2009; Diehl et al., 2014). Both subjective age and age identity have been critiqued of due such decontextualization. For example, subjective age is usually defined as the overarching answer to the question of how old an individual feels regardless of specific situations (Diehl et al., 2014). Nikander (2009, p. 865) launches similar criticism of age identity claiming it fails “to detail the interactional processes whereby positive and negative cultural meanings of age are mobilized in the multitude of immediate local contexts”. In an attempt to highlight the situational aspects that serve as a basis for exploring and problematizing the relation between age and emotions, we follow the approach of Alfredsson-Olsson and Heikkinen (2019) and Heikkinen and Alfredsson-Olsson (2020) in the use of a framework of interactionist sociology of emotions which then is combined with an interactional perspective on age. This highlights the importance of context and interaction for experiences of age. Thus, we have chosen the concept of emotionally perceived age to analyze the dancers' experiences and feelings arising on the dance floor. In this, we build on our previous study (Alfredsson-Olsson and Heikkinen, 2019) that introduced the term to conceptualize the unexpected findings demonstrating that emotions experienced during dancing could be described in terms of age. Therefore, this study aims to deepen this initial finding by focusing on emotions and age and by adding interactionist theories on age to the interactionist sociology of emotion approach.

## Theoretical Framework

### Interactionist Sociology of Emotions

This article deploys a sociology of emotion framework that conceives of emotions as biological, social and cultural phenomena (Bericat, 2016). Emotions are perceived as complex and dynamic, meaning that emotions such as anger, shame, pride or happiness should be seen as a cluster of feelings rather than a distinct emotion. In dance, for instance, “the joy of dancing” has been described to involve delight, euphoria, happiness, and positive emotional energy (Alfredsson-Olsson and Heikkinen, 2019). There is also dynamics in how emotions may be transformed, for example, how disappointment can be processed and transformed into anger to prevent an individual from drainage of energy. Emotional states are often classified in terms of valence (positive/negative), potency (strong/weak) and arousal (active/passive) (Bericat, 2016). Generally, negative feelings feel uncomfortable while positive feelings are pleasant, yet both may have important health consequences. Positive feelings, such as joy and pride usually boost sense of confidence and capability, which in turn contribute to long-term physical and mental health (Paulson, 2009). Similarly, negative feelings seem to relate to certain forms of illness and violence (Fredrickson, 1998). However, emotion sociology does not regard negative feelings as negative *per se*. Both negative and positive emotions inform us about the meaning of a situation, event, or relationship and thus help us to act and navigate in the world. In this, emotions can be perceived as constituting an important element in our social compass that facilities our orientation in the world.

This article draws specifically on the interactionist work of Hochschild (1983) and Collins (2004). Following Hochschild's perspective emphasizing the social aspects of human emotions, we use the terms emotions and feelings synonymously. Hochschild (1983) claims that emotions function as a kind of signal system by which we navigate socially. The purpose of the signal functions is to make us aware of “what is out there from where I stand”, as Hochschild (1990, p. 119) phrased it. How we interpret the meanings of “what is out there” and “where I stand” are culturally and socially dependent and negotiated in social interaction with others. According to Hochschild, emotions involve appraisal of a situation related to changes in bodily sensations, to which we respond by displaying expressive gestures or suppressing the feeling. Finally, emotions need to be articulated to name specific combinations of the appraisal, sensation changes, and expressive displays. In this, emotions, such as “joy,” “embarrassment,” or “shame” are also expressions of the social and cultural contexts that label various bodily sensations and their expressions *via* the use of commonly shared emotions vocabulary.

According to Hochschild, every situation is governed by emotional rules, and we need to handle and process our emotions in relation to these rules (Hochschild, 1983). Emotion rules stand out most clearly when we violate them, but we gradually learn through the socialization process what to feel and how to express different feelings in various situations. Emotion rules are also dependent on various social positions, such as, age, class and gender.

Typically, interactionist sociology of emotion links emotions to social relations and social interactions. Collins (2004) argues that all social interactions between two or more persons engaging in face- to-face activity with a common focus, such as a conversation or another group activity, is an interaction ritual. If the interaction ritual is successful, a shared feeling is formed and strengthened, which in turn creates emotional energy, that is, a positive feeling providing energy to act. A successful interaction ritual also creates a boundary to others by the shared focus and is characterized by a matching bodily rhythm, that is, individuals' bodies interact in unison. Two individuals dancing together in partner dance may thus potentially accomplish successful interaction rituals when they are absorbed by a common rhythm and focus on the mutual dance steps. While successful interaction rituals create emotional energy at the individual level, they also advance group solidarity. An individual's emotional energy can be replenished when engaging in new interaction rituals, if they are successful, or drained, if they fail. According to Collins (2004), social life is characterized by a constant pursuit of maximizing emotional energy, which has been termed emotional energy tropism. Individuals move from one interaction ritual to the next interaction ritual, creating chains of rituals.

Incomplete or failed interaction rituals may create negative feelings of shame, anger and exclusion, which leads to drainage of emotional energy according to Collins. Negative feelings can also originate in the non-fulfillment of an individual's expectations in a situation (Scheff, 2000; Collins, 2004; Boyns and Luery, 2015).

Collins has primarily studied positive emotions and the energy created through successful interaction rituals strengthening such feelings. Failed interaction rituals are seen as draining and are therefore something we avoid. Research has also pointed out how negative emotions, such as anger, sometimes may mobilize energy, which has been coined as negative emotional energy (Bramsen and Poder, 2014; Boyns and Luery, 2015). In this article, we do not elaborate on this matter and thus only apply Collins' concept of (positive) emotional energy.

#### Flow

During successful interaction rituals in dance, a context usually governed by emotion rules of joy, certain kinds of experiences reminding of what has been termed flow (Csikszentmihalyi, 1975/2000, 1990) seem to occur (Heikkinen and Alfredsson-Olsson, 2020). Flow has been called an optimal experience (Csikszentmihalyi, 1975/2000, 1990) that is highly pleasant and enjoyable. It has been suggested that flow is what really motivates individuals to pursue leisure activities (Stebbins, 2005; Elkington, 2011). Flow experiences may occur during high levels of emotional energy in dance and can be seen as precious in emotional energy tropism (cf. Boyns and Appelrouth, 2011; Heikkinen and Alfredsson-Olsson, 2020).

The concept of flow is included in the theoretical framework in this article because it clarifies the transcendental character of some of the experiences during high levels of emotional energy in dance (see also Heikkinen and Alfredsson-Olsson, 2020). Flow was coined in by Csikszentmihalyi (1975/2000) in the 1970s to name the autotelic experience, i.e., an experience which is highly enjoyable and intrinsically rewarding. It is a psychological state in which awareness and action merge while we focus solely on the activity itself forgetting about irrelevant stimuli and external concerns. We have a sense of control even if we lose self-consciousness and time is transformed. Flow may occur in activities that are goal-oriented, require skills that are balanced in relation to the challenges, and offer immediate clear feedback.

In the pioneering work on flow by Csikszentmihalyi (1975/2000), one of the empirically investigated activities was dance to pop and rock music. Csikszentmihalyi points out that the dance seemed to involve opportunities of a social kind which were not investigated in the study. Nevertheless, he exemplifies how “some dancers focus on interaction with their partners. The partner serves as a source of feedback to the subject's dancing and self-expression” (Csikszentmihalyi, 1975/2000, p. 105). In the context of the couple dance investigated in this article, this kind of social feedback may be of highest importance for flow since the partners are even holding each other as a contrast to rock dancing where the dancers do not touch each other and may concentrate on individual expressive movements to music.

### Interactionist Framework on Age

The interactionist framework is also applied in this article to the way we conceptualize age. In this, age is enacted and (re)created in social interactions (Alanen, 1988; Laz, 1998; Calasanti, 2003). Age is relational, entailing that, for instance, age as a category is always constructed through social processes in relation to other age categories, and if one age category changes its meaning, then all other age categories change as well (cf. Emirbayer, 1997). Age is something that we all do or rather the meaning assigned to age is continuously (re)produced in various social situations (Alanen, 1988; Laz, 1998; Calasanti, 2003). Age thus becomes an important dimension in how we perceive situations, how we act and consequently, from Hochschild's perspective, how and what we feel.

Age has been described as a flexible category as it relates to the manifold positions of the human life course from birth to death. Regardless of age, individuals can often switch between describing themselves as young or old. Sometimes chronological points in time also play an important role, for example, certain birthday years such as 50 years can be celebrated in various cultures (Nikander, 2000, 2009). A great deal of age practices and doings are formed around two opposing poles: “young”/”younger” and “old”/”older”, related to which age span is normative in a social context. This means that in a context when most people are older than yourself, you may consider yourself young regardless of your chronological age and vice versa (Nikander, 2009). However, while age is being a flexible category it is similarly to emotions also experienced in and through the body. Age is embodied (Laz, 2003). Bodily sensations can at times remind us about our physicality and may be interpreted as age, while at other occasions, the body goes rather unremarked. Bodies also change over time and thus may change our experiences. The interpretations and understandings of the body draw on cultural resources and interactional encounters, making bodily aspects such as fitness, health, illness and energy often understood in relation to age (Laz, 2003).

There are certain social and cultural ascriptions that define some ages as less desirable than others. For example, “old age” is a particularly unfavorable category that is associated with decline, misery and loss in Western cultures, while young age is a desirable category connected to beauty, vitality, and strength (e.g., Gullette, 2004). The practices of doing age are therefore also related to the instances of power enactments aiming at maintaining the system of privilege afforded to younger ages. For instance, the temporality and choreography among younger social dancers are usually considered as “the normal”, and older people's social dance is marked as deviance (Krekula et al., 2017). Age is an important category that is used to differentiate, order and rank, and through this, age contributes to the system of inequality reproduction (Krekula et al., 2018).

In this construct, age is a cultural resource that, like any other category, helps us make sense of reality, but also limits our way of thinking (Juhila, 2004). For example, when 55+ members of the University of the Third Age (joint educational, social, and recreational programs addressed to senior adults U3A) in Poland reject the idea of old age, they reject the socio-cultural construct that has also very strong moral underpinnings (Wilinska, 2012). In this, age provides a moral compass by differentiating between age appropriate and age-inappropriate actions (Laz, 1998), and thus also sanctions and praises giving raise to emotions such as shame and pride. This points to the high level of emotionality of age-related practices.

Age can also be understood in terms of various age identities and as such it relates to a sociological tradition of social norms, age categories and role theories (Diehl et al., 2014). This is the way we use the concept of age identity in this article. Based on Goffman, identity can be seen as “*the subjective sense of his own situation and his own continuity and character that an individual comes to obtain as a result of his various social experiences*” (1963, p. 108). Sense of identity is created through experiences of (non)identification with social categories, roles, and social meanings. This also entails that individuals can develop a certain degree of a role distance. For example, while they may invest in acting and dressing youthfully, they may not necessarily identify as young people. Age identity can thus be viewed in some degree as continuous and stable aspect of the self while being formed by numerous social encounters and experiences (Kaufman and Elder, 2002). Moreover, we are highly sensitive to others' assessments of how we act or who we are, giving rise to shame and embarrassment (Goffman, 1963), or pride and shame (Cooley, 1902/1972).

Building on interactionist frameworks, this article focuses on emotions and age. We propose that in addition to serving the purpose of justifying everyday choices and decisions and accompanying the process of identity-creation and identifications, age is effectively used to speak about and account for different emotions.

## Materials and Methods

This study is part of an exploratory and ethnographic research project on the social aspects of dance in old age, funded by the Swedish Foundation for Humanities and Social Sciences (P15-05754:1). The data material was collected in 2016 by four researchers participating in 14 different dance events, conducting qualitative interviews with 44 dancers and/or organizers of dance events for the older people and analyzing documents on the various dances. Various parts of the material have previously been analyzed, i.e., the organizing of dance for older people (Krekula et al., 2017), emotions and the interaction of emotional energies (Alfredsson-Olsson and Heikkinen, 2019; Heikkinen and Alfredsson-Olsson, 2020), temporality in senior dance (Krekula, 2020), and dancing through life (Heikkinen, 2021).

This study is based on the interviews with older persons involved in social dancing, i.e., the dancing by “going out” at public dance venues (see also Heikkinen, 2021). The interviewees were recruited in connection with participant observations at various dance events. Since some interviewees were also engaged in other types of dances beyond social dance, they were recruited both at social dance events as well as other events. More specifically, the interviewees were recruited from a folk park (a kind of public recreation park), a dance festival, a folk-dance group, dance events organized by retirement organizations, dance on a boat cruise, dance at a fitness center and artistic dance. Some dance events specifically targeted older people while others aimed at a broader age range, even though many dancers were older. The dance events that were specifically arranged for older people had the age limit of 50 years and, thus, we chose to adopt the age of 50 as the minimum age when recruiting the interviewees.

The sampling strategy was a combination of convenience sampling and purposive sampling. It was convenience sampling based on who the researchers talked and danced with at the dance events. It was purposive sampling aimed to achieve variation in age, gender, and other social characteristics (e.g., from beginner to experienced dancers). The project group met regularly to discuss sampling in connection with dance events observations.

The study involved 29 interviewees, of which 26 were still engaged in social dance while 3 had quit recently. All participants had experiences of social dance in terms of partner dancing, a few had also visited a senior disco (for further information on types of dances for older people in Sweden, see Heikkinen, 2021). The age span was 53–82 years with a median age of 72. The majority were women, 20 women and 9 men, which reflected the age distribution at social dance events for older people. The semi-structured interview guide was constructed and discussed within the project group. The themes were broad and included, for example, emotions and relationships, intimacy, dancing through life, experience of age, and norms and transgression of norms. Each theme included several suggestions on follow up questions while the interviewer was free to ask new questions depending on the character of the interview. The participatory visits on dance venues meant that questions related to specific dances could be included. We noticed early in the project that many individuals were very positive and excited about their dancing and therefore deliberately included questions about negative emotions in order to get beyond “the joy of dancing”. Moreover, we had questions about situations when the interviewees felt old and when they felt young. Each interview lasted between 1 and 2 h.

The interviews were recorded and transcribed. The material was analyzed thematically (Braun and Clarke, 2006). We read the interviews and coded through making marginal notes when the interviewees described themselves as young/younger, old/older, their own age, and when age did not matter. Then we marked the emotions described and evoked in these contexts, upon which a new coding was made based on the questions regarding why feelings arose and why interviewees highlighted feelings of age or feelings related to age. This step generated codes such as flow, other dancers' age, dance partner's age, attraction, body, and skills. Based on our analysis, we could identify first, how dancing as an autotelic activity contributed to the individual's experiences that age did not matter, second, that the perceptions of age of other dancers nevertheless did make age matter, and finally, how the experiences of one's own body and skills evoked feelings and thoughts about age. The following result sections are organized around these three themes of dance. All interviewees, geographical places and names of dance events have been anonymized. All translations of quotations from Swedish to English have been checked by a translator. The study complies with the ethical requirements of the Swedish Research Council.

## Results

The results are presented through three themes. The first theme analyzes the interviewees' claims that age does not matter when dancing. The second theme highlights that, nevertheless, age does matter in certain situations. Age becomes visible in relation to other dancers' ages and may bring forth socially relevant norms of age and gender. The third theme, finally, shows how the dancer's body with its possibilities and limitations evokes feelings of age and how the bodily felt emotional energy in itself may be denoted as an emotion of age.

### Age Does Not Matter When Dancing

A recurring utterance among the interviewees was that age does not matter significantly in dancing. This relates to the way dance is practiced as an intrinsic rewarding leisure activity and how dancing brings positive emotional energy and occasionally flow experiences (cf. Hefferon and Ollis, 2008; Alfredsson-Olsson and Heikkinen, 2019; Heikkinen and Alfredsson-Olsson, 2020). In this empirical section, we examine aspects which contribute to the interviewees' claims that age is irrelevant and that they did not think about their age when dancing.

One main feature of dancing for most of our interviewees was that they practiced it as an autotelic experience, they danced for the sake of dancing. Dance is an activity of pleasure, as illustrated below:

Interviewer: Is that why you dance, for health reasons? Or why do people dance?

Anders: I dance because it's so much fun and so enjoyable. When you've reached the stage of really mastering it, then it's always the same divine feeling. Then it's obvious that I get positive reactions that have to do with dancing, it's great fun all of it. Overall, that's the point of it all. (Anders, 69)

Interviewees repeatedly emphasized various positive feelings attached to dancing, something that has been named “the joy of dancing”. This includes emotions such as delight, euphoria, and happiness (Alfredsson-Olsson and Heikkinen, 2019). Similar to Anders, the majority of the interviewees emphasized that they primarily visited dance events to dance instead of extrinsic reasons such as meeting a new life partner or establishing social bonds outside of the dance arena. The characteristic of dance as an autotelic activity can be understood as downplaying the ways age matters, for example, in relation to social norms of partnership. The joy they experienced when dancing allowed the interviewees to participate in a successful interaction ritual that strengthened the emotions of joy and brough emotional energy. Stebbins (2006) asserts that leisure activities, such as dance, generally are rewarding, because they are voluntary and people can choose which activities to engage with to replenish their emotional energy.

Autotelic experience has been claimed to be a main feature of flow (Csikszentmihalyi, 1975/2000, 1990; Stebbins, 2005, cf. Elkington, 2011). We can also see that the participants in this study occasionally experienced what can be interpreted as flow, or as Anna recounted it:

When you get into that slow feeling. It's not in every dance and not in every tenth dance either. But it is a certain state of being you get into when you feel really good. (Anna, 65)

Flow can be described as a temporary state when the interaction rituals are performed smoothly and the emotional energy levels are very high (cf. Boyns and Appelrouth, 2011; Heikkinen and Alfredsson-Olsson, 2020). According to Stebbins (2005), flow is a main drive for practicing leisure activities due to its highly rewarding character. Using Collins' (2004) vocabulary, this can be also understood in terms of emotional energy tropism that explicates practices of orienting oneself toward situations that may increase the levels of emotional energy, for instance striving for high emotional energy experiences such as flow (Boyns and Appelrouth, 2011; Heikkinen and Alfredsson-Olsson, 2020). Expressions pointing to temporary experiences of flow such as “finding the good feeling,” “it flows,” “hover,” and “floating” were repeatedly used by the interviewees, or as Anna and Agneta put it:

What you feel when dancing, it's like the world disappears. You're over the moon. I can't describe it. Like an energy kick. (Anna, 65)But finding the very special hallelujah-feeling, you know, when you get, well, we would say, “we fall maddeningly in love”. (Agneta, 53)

While flow is being a highly enjoyable experience, which may be added to the cluster of positive feelings forming “the joy of dancing” (Heikkinen and Alfredsson-Olsson, 2020), flow also includes perceptions of altered reality and consciousness (Csikszentmihalyi, 1975/2000, 1990). This means that awareness of social categories, for example those based on age, can be understood as temporarily interrupted in the dance. Above, Anna, for example, talks about how “the world disappears”, which signals transformation of time and space as well as loss of self-consciousness (Csikszentmihalyi, 1975/2000, 1990). Also, other aspects that are culturally associated with old age, such as pain (Laz, 2003), can be forgotten in flow experiences. Here is Lisa's account of what happens with the body in the dance:

I can't add anything more about what's so great about it [the dance]. But then moving to music is wonderful, the body produces endorphins, you don't feel the body…aching knees and joints when you are dancing. You do, though, the next day. (Lisa, 68)

In other words, flow experiences can be assumed to weaken the role of age in the dance as they result in, at least temporary, a lack of awareness of age. During the state of the high emotional energy of flow, the older dancers are not reflecting upon age, which can be one explanation of the recurring claims that age does not matter when dancing.

Another instance in which the irrelevance of age was brought up by the interviewees concerned the partner's age. In partner dancing, the core activity is interaction rituals with only one partner, which means that dancing with the right person is important. Emotional energy can only be gained as a collaborative effort between the dancers. Johanna expressed this as follows:

You have the experience of dancing together, feeling the music and rhythm and, well, it's special because you can't experience that with anyone on the dance floor, but it's something, well, you must find it. And it has nothing to do with someone your own age—it could be someone younger or older. So that doesn't matter. And looks don't matter either when you feel that special connection. (Johanna, 79)

The quotation illustrates important aspects of successful interaction rituals (Collins, 2004). The dancers are dancing together and “feeling the music and the rhythm”, which signals corporeal synchronicity. The togetherness of the experience points to a shared perception of the situation and presumably a shared overall feeling of joy. The dancing couple constitutes a distinct unit with boundaries to other couples. For this experience of togetherness, the age of the partner seemed to not matter. In a similar vein, a number of other interviewees underlined that having a good dance experience did not depend on age. Agneta claimed, for example, that dancing was “ageless”, saying that it made no difference to her if her dance partners were “22 or 72 years old”. Instead the suitable dance partner was described in terms of an accomplished dancer, or as Eivor and Elsa said:

Is he a good dancer? If so, age is of no relevance, not for me. And you sit there observing and checking, well he seems like a good dancer. (Eivor, 75)

It is still this...well, how can I describe this special feeling of... it's a combination of music and movement that sort of makes... You know, I don't really know how the body functions, but it's euphoric. Then, as I've always said, if I'm at a dance, let's say I was forty-fifty, and there was a man, 80 years old, who was the best dancer, then I would ask him to dance with me because I love to dance. (Elsa, 72)

Eivor's account illustrates the selectivity apparent in the interview material related to the partner. This importance of finding the right partner can also be related to flow theory. In partner dance, the requirements of clear feedback as well as, it can be presumed, the balance between skills and challenges, are highly dependent on who you dance with (Csikszentmihalyi, 1975/2000). Thus, also in order to reach the specific moments of high emotional energy characterized by flow, you have to find the right partner, in this, age have little significance.

Above, we have illustrated how partner dancing provides experiences of flow, during which the interviewees neither feel nor are aware of age. We have also showed that the importance of age diminishes since the partner's age seems unimportant in situations when the interviewees seek emotional energy and ultimately flow experiences. In this, dancing was also termed “ageless”. The focus is on finding a partner with the right skills and matching dancing style to generate positive emotional energy and flow experiences.

### Other Dancers' Age Evokes Feelings of Age

Although the participants claimed that age was irrelevant, references to age were frequent during the interviews. For example, age became emphasized when the interviewees referred to age differences between dance partners or dancers in a particular dance event. In this section, we investigate how the other dancers' age, especially the dance partner's age, can contribute to feelings of being young(er) or old(er).

Age is relational and the experience of one's age is directly affected by the perception of other persons' age in a context (cf. Tsui and O'Reilly, 1989; Calasanti, 2003; Avery et al., 2007). This is notable in the interview material. One example is the account of Doris, 68 years old, who said that if she would go to a disco or nightclub with young people, she would feel old and “people would probably look at me and wonder if grandma had arrived”. She explained that if, on the other hand, she went to a dance with a mixed crowd, she would not “feel like a lost person”. Agneta described similar experiences:

That's when [I think about age]. I see that maybe 75 percent of people there are younger persons. Then I wonder if this [the dance] is for us anymore. But at the same time, for heaven's sake, we have fun. And there is a couple as old as we are! And that's enough somehow! (Agneta, 53)

The age of the dancers present at a dance event creates a normative age. Obvious deviations from the norm give raise to the experiences that are termed, as in the examples above, feeling or being old. Emotions of alienation emerge here, such as in Doris' way of talking about “being lost” and Agneta's question if dance is still for her and her partner. The experience of feeling old is both related to one's own assessments of not fitting in and, as shown in Doris' statement, to the gaze of others. The feeling of alienation made our interviewees avoid dances that they perceived to be youth dances and instead they chose mixed dances or senior dances.

When visiting dance events, the significance of age also emerges in relation to age differences between the two dancers in partner dance. Even if the interviewees claimed that the age of the partner did not matter, cultural norms of acceptable age difference intervened in the dance for both women and men. For women, the violation of norms mostly seemed to be an odd or even comical instance. For example, Eva, aged 73, remembered how her female friend “laughed her head off” when Eva was asked to dance by a few men in their twenties at a dance event. However, the reverse situation involving older women asking out younger men to dance appears to be more problematic:

I don't ask a 40-year-old to dance. I don't believe he would be very happy to dance with a 70-year-old crone (Lisa, 68).

The use of the word “crone” to describe oneself in relation to a younger man signifies a moral norm attached to the relations between men and women. Even if younger men can ask older women to teach them to dance, it is not appropriate for 70-year-old women to ask young men for a dance. In a similar vein, the rules regarding older men and their prospects of dancing with younger women are also clear.

Because I don't want to dance with young women, but with women in their 40-50-60ies. But it happens, you know, that very young women dance with men aged 60-70. The age span might be 3040 years. If this happens, you get odd glances although you are there to [dance]... yes. I know a few who have this label. /…/ Then you might think, dance with someone your own age too. If you get that label, you might feel haunted by it. (Gunnar, 68)

The inappropriateness of dancing with a much younger woman is here presented in terms of a situation which is potentially stigmatizing. In this case, cultural stereotypes of “dirty old men” seem to be in play referring to inappropriate sexual drives among older men (Walz, 2002). Being viewed through this kind of stereotype is both a “tribal stigma” related to one undesired social category and to deviations in personal traits (Goffman, 1990). Other interviewees, for example Einar, used expressions in Swedish which are close to the English expression “dirty old men” (Swe. “ful fisk,” “snuskhummer”) and Einar talked explicitly of shame when describing the situation of being viewed to dance with women too young. Appropriate and inappropriate age differences are an issue here (Laz, 2003). Gunnar's statement above elaborates on the different age spans and their appropriateness and inappropriateness and his concern about violating the norms, indicating the delicate balancing act involved.

Concurrently, however, another pattern arises in the interview data which shows that dancing with a younger person (within the appropriate age span) is something positive and contributes to feelings of being young, as shown in the following quotation:

[You get young] when you dance with a young woman and she's laughing and happy and wants to and asks you to dance again. If we get to dance several dances, you feel young. Happy and young, then your spirits rise immediately and then you feel young (Einar, 78)

The quotation suggests that Einar feels attractive and confirmed by a desirable person, as this person belongs to the culturally valued category of young (Bytheway, 1995). This creates a feeling of pride (Cooley, 1902/1972). He also is allowed to participate in the interaction rituals of dance, which contributes to emotional energy. He feels uplifted and happy and continues engaging in the interaction ritual of dance. Also, women may become proud when they are asked to dance by younger men. For example, Elisabeth, aged 63, talked about a younger acquaintance who “came and asked me to dance. And then I actually got a little honored”.

The lack of response and confirmation from a younger person can, on the other hand, shape an emotion labeled as feeling old. Einar expressed as follows:

I feel [old] sometimes when I ask a young woman to dance who I think is good-looking and zesty and all that, and maybe not like me, considerably younger than me, and if she's not attracted to me and I get no response, then I feel old, actually. If I see that she, for instance, looks in another direction when I'm heading towards her, then I feel old (Einar, 78)

The lack of confirmation from a desirable person results in what may be assumed as a cluster of negative feelings. Being rejected means not being able to participate in the interaction ritual as intended, something which drains emotional energy. Since it is a younger person who acts in that way, Einar relates it to his age and labels the emotion in terms of old. Women have similar experiences of being ignored, which creates feelings of unattractiveness and feelings of being old. This is evident in Elsa's account:

Let's say that I was one of the better dancers, and still don't get asked to dance, because I'm older. Men would rather dance with younger persons even if they are poor dancers. And that is, then I don't feel like going out. /.../ And then I can feel that now age takes its toll. /…/ then I end up further and further towards the back and finally I'm a wallflower. I hate those dances, I don't really like to go there at all. /…/You feel older than your age. You lose confidence going to such a place if there's a risk that you won't get to dance. (Elsa, 72)

In this case, however, it is not a matter of a younger man opting out of dancing with her, but of her not being allowed to dance since men prefer to ask younger women. Her wish to dance is not granted even though she is a good dancer. In Elsa's view, her ability to dance is not the issue, but rather that her age makes her unattractive (cf. Hurd-Clarke and Griffin, 2008). The quotation highlights the experience of being rejected and excluded from the dance. In becoming “a wallflower”, Elsa misses the opportunity for interaction rituals and emotional energy. She then almost loses her “self-confidence”. The extract thus points to how feeling old in this case derives from negative emotions related to feeling unattractive, discarded and not gaining access to emotional energy by dancing. However, the feeling of being old is only temporary.

Because I'm relatively fit, I feel fairly young. I thought I would feel older at 72 than I do. I think so at least. And my husband too. So, we are pretty privileged. We don't have any aches and pains either, now. /…/ Well, the only time I felt old is, as I said, when you are at a dance like that [as described in the quotation above]/…/it's a kind of dread, a fear I have of, like, that this will happen, you know [referring to a situation when she was not asked to dance] /…/ I don't have to go to those dances in that case. There are other dances. (Elsa, 72)

The quotation above can be understood in terms of adopting role distance (Goffman, 1963) to the dance event when she felt old. Role distance is marked by words such as “the only time”, suggesting it was only a temporary role she was forced into. Although presenting a younger identity than her chronological age, she is nevertheless threatened by the idea of not being asked to dance. Identity is formed by constant social encounters, and in this case, Elsa preserves her identity as young by opting out of certain dances.

Above, we have shown that age norms, both those created in the situation by others present at the dance and cultural age norms, impact on the dance and disturb the sense of “agelessness”. These age norms are evoked through age differences among the dancers and shape feelings of belonging, alienation, pride and shame. Opportunities, or lack of them, to participate in the interaction rituals and gain emotional energy also affect the emotions evoked, which then, together with the emotional energy level, are labeled and experienced as age. It should also be pointed out that there is usually a surplus of women at the dances, especially senior dances. The experience of being attractive or not, being rejected or not, and the opportunities to participate in the interaction rituals are thus gendered (see also Alfredsson-Olsson and Heikkinen, 2019).

### Dancer's Own Body Evokes Feelings of Age

The social constructions and experiences of age have also their bodily grounds. Thus, even though dancing can diminish some bodily sensations such as pain, especially when experiencing flow, the body comes to the forefront on other occasions. To a great extent, dance is a bodily activity, involving dancers and their physical abilities. Experiencing failure due to bodily limitations or accomplishing physically challenging dance steps are associated with age. In a similar vein, experiences of emotional energy are firmly grounded in bodily sensations which can be related to perceptions of younger or older ages. The present section discusses these aspects of old age, dance and emotions.

Recurrently, feelings of age in our interviews were expressed in utterances such as “I can still do it” and “I have sort of tested myself”. Being able to perform difficult steps or failing to do so are related to talk about feeling young or old. For example, Einar remembered dancing with a woman whom he considered both young and accomplished as a dancer:

She danced nearly like a night club dancer [chuckle]. A bit too much on the hips … oh … ooh… and I tried to keep a high tempo and my ankles hurt, I was tired. And then I danced with another that I usually danced with, and my ankles were tired, you feel... I feel that age takes its toll... (Einar, 78)

The experiences of feeling old are clearly related to the ways in which age and body are mutually dependent on each other. Laz (2003) explains that relationship in terms of age embodiment that highlights the importance of body to the ways in which age can be experienced. Embodiment, the physicality of our social identities, sometimes goes unnoticed while at other occasions being highly salient (Laz, 2003). In the situation described by Einar, his body fails him. The tiredness and ache become reminders of his bodily limitations that he interprets and experiences as feeling old, emphasizing that “age takes its toll”. In contrast, the ability to follow complicated dance steps and high pace of dance can contribute to feeling young.

Interviewer: Please tell us about a dance occasion when you felt young and what it was that made you feel young.Doris: Well, I think that happens when you keep up with the quick tempo music in Jive for instance. I can't mention a specific occasion, but... But when you can swing about a bit, I think... I don't feel old. [chuckle] I never feel old at all. (Doris, 68)

Typically, high-paced dances, such as jive are presented as an arena where Doris tests her bodily capacity. Feeling young in this case is associated with the ability to move fast and to follow. There are thus the situational aspects related to a specific dance style that suggest a capable body, which in turn generates the overall feeling of being young, or rather feeling of “not being old”. The negation used by the interviewee can be seen as a vivid reminder of images and expectations associated with certain bodies. Old bodies are often not perceived as fast and while performing dance that requires speed and flexibility, Doris actively negates such images, showing that her body does not fit the stereotype (Gullette, 2004). Kristina, aged 72, also talked about how she feels young when performing difficult steps in jitterbug. However, she quickly explained that feeling young is “not entirely fun... I think that I feel fine about my age. I'm satisfied with my age. I'm 72 and value it, and I don't wish to be young again”. Thus, Kristina makes a distinction between feeling young in the very moment of dancing and the age she wants to be and identifies with, indicating that the relationship between feelings of age and age identity is not straightforward (Goffman, 1963).

Fitness is associated with youth and some of the dancers pointed to the fact that they are physically fit as a reason for feeling young when dancing. Anna, 65 years old, noted that dance has given her “a basic fitness” and that she has “stamina” and thinks she has “become younger” by dancing. This feeling becomes strengthened through comparisons with younger dancers. For example, Berit aged 82, who considered herself fit and said that her physicians thought she was much younger, explained that “there are many young people who look old” at dance events and this depends on “how they take care of their own bodies”. By both pointing to other persons' affirmation of her as younger, an important influence upon the self (Cooley, 1902/1972), and stressing that many unfit younger people look old, she conveys a more stable identity as younger than her chronological age based on bodily appearance and fitness. At the same time, the comment related to taking care of one's body emphasizes individual responsibility and the idea of body as a project that can be changed and shaped according to one's preferences (Laz, 2003).

Furthermore, emotional energy which is grounded in the body and has been described as an individuals' inner source of energy seems also to be interpreted in terms of age. Frida remembered a dance experience:

But it was heaven to dance with him, I just flew around, floating on clouds, and then I bloody well felt like 15 years old I think…(Frida, 74)

The high level of emotional energy is reported here in terms of feeling like being 15 years old. However, several words such as “bloody well, like, I think” suggest some kind of distance and point to that she might not really identify as 15 years old. Still, she needs this description of age to mediate the sensations she experiences in her body. A similar connection between high levels of emotional energy and feeling young was found also in other interviews. Klas, 73 years old, talked about how he felt young after an extraordinary good dance with a lady of his age. The dance gave him “a proper energy boost” and he felt happy. Anna also elaborated on when she has felt young in the dance:

Interviewer: Can you tell me about an occasion when you felt young when dancing?

Anna: On every occasion... because you get a special feeling in your body when dancing. I can feel that the body is hanging in there and I don't have aches and pains anywhere. I broke my foot quite badly and it took one year before I could walk without a limp. But if I went dancing it didn't take more than 2-3 dances and then it was gone. I felt no pain—it disappeared. So, it has something to do with endorphins, they make you happy and if you are happy, you feel young and fresh. I think it's in this dimension. (Anna, 65)

In response to a question about feeling young, Anna mentioned how her experience is clearly related to what she terms “a special feeling in your body”. The experience is about lack of pain, being happy and presumably having high emotional energy as she seems to satisfactorily participate in the interaction rituals of dance. The body evokes sensations which are interpreted as age since lack of pain and being energetic are culturally connected with youth (Gullette, 2004). Similarly to Klas, happiness is associated with age, an association which can be understood as a cluster of both a bodily grounded high level of emotional energy and happiness. Correspondingly, low levels of energy are related to tiredness, lack of enjoyment and feeling old, or as Einar put it:

It's sort of shameful, have I reached such an old age now that… that I am an oldster, I cannot do it anymore, and you sort of feel it, and you try to master it but… and I have to slow down and rest for a couple of dances and sit down on a chair for a while, because…I felt it last Thursday in particular… that I… it is only this last year that I have felt it. You actually get tired… so… it's not a good feeling either…You are reminded that you are old (Einar, 78)

The body prevents Einar from entering situations that could lead to an increase in emotional energy. Instead, any attempt at dancing becomes draining and exhausting. There are these experiences of tiredness that not only underline the significance of the body, but, above all else, the significance of old body and its limitations. In sum, this makes Einar feel old. As it is emphasized in the excerpt, feeling old is not a positive feeling. Old is associated with qualities that Einar would not like to identify with. In this, the excerpt provides an illustration of a complicated relation between emotional energy, old age, body, and identity. Describing this in terms of “shame” becomes a vivid reminder of norms attached to old age (Laz, 2003; Gullette, 2004).

In this section, we have shown how feeling young and/or old are deeply embodied. The physicality of dancing combined with physicality of aging creates a context in which older dancers frequently become reminded about their bodies, both their bodily capacities and limitations. This intensified focus on their own body among other bodies evokes strong emotional responses that seem to go beyond the regular emotion vocabulary. Instead, notions of feeling young are being used to denote high emotional energy levels as youth is culturally associated with being energetic (Gullette, 2004). In a similar vein, the dancers feel and are clearly aware of occasions of low levels of emotional energy. In the socio-cultural context, where aging is associated with decline and limitations (Bytheway, 1995; Gullette, 2004), those low energy levels become transferred into the feelings of being old.

## Discussion and Conclusion

This article deepens the understanding of how age as a highly social and cultural phenomenon is, in certain contexts, effectively employed to denote emotions. We have used the concept of emotionally perceived age to highlight the application of an interactionist sociology of emotion framework, combined with concepts and ideas from an interactionist perspective on age. In this way, the article brings out the understudied topic of the situatedness in which people talk about feelings of age (Nikander, 2009; Hughes and Touron, 2021).

The two research questions which guided this study were: (1) how emotions are evoked in relation to age in dancing, and (2) when and how age is described as a feeling. With regard to the first research question, we have discussed three primary aspects of evoking emotions related to age when dancing. The first aspect calls attention to the specific characteristics of dance as a leisure activity that weakens the importance of age. Successful interaction rituals in dance lead to positive emotional energy (Collins, 2004) and ultimately even to flow experiences (Csikszentmihalyi, 1975/2000, 1990). The emotions exposed in this context are those characterized by “the joy of dancing”, namely the cluster of high levels of emotional energy, happiness, euphoria, delight, and occasional flow (see also Alfredsson-Olsson and Heikkinen, 2019) which contributes to older dancers “forgetting” about age. Moreover, the partner's age is claimed to be not relevant when dancing, since the success of interaction rituals is dependent on the partner's skills and dance style rather than age.

The second aspect focuses on how age differences among the other dancers nevertheless do make age a prominent feature in dance and, in turn, evoke emotions and feelings of age in several ways. Deviation from the age norm at dance events may awaken feelings of alienation which then may be termed as feeling old. Similar findings have been described in the field of relational demography on age and working life (see e.g., Tsui and O'Reilly, 1989; Avery et al., 2007). This strand of research explicates how the age distribution at workplaces, in working groups or between colleagues raises a range of emotions. This article shows that even apparent age differences emerging during short periods of time and within specific places can contribute to emotions related to age. Thus, for example, the feeling of alienation as a consequence of belonging to the wrong age category at a dance event can immediately be termed as feelings of age.

The prominence of age is noteworthy also during situations involving dance partners whose ages significantly differ. To dance with a young person that belongs to a socially valued category may therefore create a sense of pride and joy, which in turn is often described by the research participants as feeling young. By the same token, to be ignored by a younger person can lead to feeling old. However, cultural norms of inappropriate age differences may intrude if the age differences are too big, raising feelings of shame, especially among men who risk being stereotyped as “dirty old men” (Walz, 2002).

The third aspect emphasizes the bodily basis of emotions related to feelings of young and/or old. Bodily limitations and failure to perform difficult steps relate to being tired, emotional energy drainage and may be termed as feeling old. In contrast, bodily fitness and success in performing difficult steps that enable participation in interaction rituals and create emotional energy may be highlighted as feeling young. There seems to be a clear association between how very low emotional energy levels or very high emotional energy levels can be labeled as feelings of age.

The second research question narrows the focus to how and when emotions are denoted as age. This means that here, we are not interested in how different emotions are evoked in different situations at the dance, but rather in how and when they may be attributed to age. Age differences and bodily sensations give rise to different kinds of emotions, which sometimes becomes feelings of age because the original emotion is attributed to be caused by age. For example, alienation may become experienced as feeling old in the emotional dynamism of changing feelings (Bericat, 2016) when an older dancer tries to figure out what this emotion is at a dance venue with mostly younger dancers. Appraisal or devaluation of someone from a different age group may be attributed to one's own age, and the resulting feelings of pride or shame may therefore be assigned to age. However, what is surprising is the way some emotions or bodily sensations seemed to be felt like age quite directly. Low levels of emotional energy, tiredness and pain were talked about as feelings of old with little concern of situational aspects such as who the dance partner was or how good the dancing had been. High levels of emotional energy in successful interaction rituals appeared to make age less relevant and in the case of flow even set it aside temporarily. Occasionally, however, those moments were termed as feeling young. One interpretation is that the disappearance of pain during flow means that the awareness of the aging body diminishes which then is termed as the contrast to old, namely young. Another interpretation is that these kinds of highly intensive emotional moments may be difficult to verbalize, which in some occasions give rise to the usage of age categories that due to their cultural connotations may help to convey such feelings. Overall, age categories seem to be important instruments to convey feelings without necessarily concerning identification of being a certain age. In this, similarly to how we talk about other emotions such as anger and happiness, age becomes a recognized feeling whose emotional character we somewhat understand. This points to the manifold ways age manifests in our culture, including its use as a feeling.

A few studies point to similar results that energy levels and even specific emotions are felt like age. Even though Choi et al. (2021) focused on a general feeling of age (not situational), they found that low levels of energy were related to feeling older than one's chronological age. Dutt and Wahl (2017) showed how sadness, which often is accompanied with low emotional energy levels (Collins, 2004), induced feelings of a higher chronological age. However, we know little about these kinds of emotional aspects of age. Interactionist emotional frameworks can be fruitful in order to explore how and when emotions become feelings of age as these can capture emotional dynamics. There is still little knowledge on situational aspects of feelings of age (Hughes and Touron, 2021) and to our knowledge, this matter is rarely explored by utilizing an emotion sociological framework. We also argue that dance may be an important context to explore emotionally perceived age as the corporality of age and dance seem to enhance each other creating very powerful feelings, which may more clearly illuminate processes contributing to feelings of age.

One important finding in the article is that age is used to label feelings without necessarily concerning identification in Goffman's (1963) sense. This was one of the reasons for us to use the concept of emotionally perceived age instead of age identity. The empirical material discussed in this article shows that the frame of reference often does not concern issues of identity, but rather concerns usage of a vocabulary that allows to fully express what one feels while dancing. Age becomes a useful category to name and assess situations, bodily sensations, and actions.

This article contributes to research discussions regarding feelings of age and the ways these are created interactionally by focusing on older people's dance. Our examples and discussions presented in this empirical and explorative article demonstrate a great variation in the ways in which feelings of age may be evoked and experienced. As such, feelings of age are not about inner states following people's concerns over their ages but are rather collaboratively created in the process of social interaction. In this, the article expands research on aging and dance by illustrating the complexity in how emotions are formed in interactions at dance events and how dancing may contribute to different feelings of age. The finding of how age seems to be used to denote emotions in dance without being about identification begs question for further research about the ways we experience age. More generally, the article contributes to the research discussions on aging by highlighting the importance of context to subjective age and age identities while bringing emotional aspects to the forefront.

## Data Availability Statement

The raw data supporting the conclusions of this article will be made available by the authors, without undue reservation.

## Ethics Statement

Ethical review and approval was not required for the study on human participants in accordance with the local legislation and institutional requirements. Written informed consent for participation was not required for this study in accordance with the national legislation and the institutional requirements.

## Author Contributions

All authors listed have made a substantial, direct, and intellectual contribution to the work and approved it for publication.

## Funding

This study was supported by Swedish Foundation for Humanities and Social Sciences (P15-05754:1).

## Conflict of Interest

The authors declare that the research was conducted in the absence of any commercial or financial relationships that could be construed as a potential conflict of interest.

## Publisher's Note

All claims expressed in this article are solely those of the authors and do not necessarily represent those of their affiliated organizations, or those of the publisher, the editors and the reviewers. Any product that may be evaluated in this article, or claim that may be made by its manufacturer, is not guaranteed or endorsed by the publisher.
